# The Aging Risk and Atherosclerosis: A Fresh Look at Arterial Homeostasis

**DOI:** 10.3389/fgene.2017.00216

**Published:** 2017-12-14

**Authors:** Trajen Head, Sylvia Daunert, Pascal J. Goldschmidt-Clermont

**Affiliations:** ^1^Department of Biochemistry and Molecular Biology, University of Miami Miller School of Medicine, Miami, FL, United States; ^2^Division of Cardiology, University of Miami Miller School of Medicine, Miami, FL, United States

**Keywords:** atherosclerosis, cardiovascular disease, vascular repair, arterial homeostasis, aging, risk factors, bone marrow-derived vascular progenitor cells

## Abstract

A considerable volume of research over the last decade has focused on understanding the fundamental mechanisms for the progression of atherosclerosis—the underlying cause for the vast majority of all cardiovascular (CVD)-related complications. Aging is the dominant risk factor for clinically significant atherosclerotic lesion formation, yet the heightened impact of aging on the disease is not accounted for by changes in traditional risk factors, such as lack of physical activity, smoking, hypertension, hyperlipidemia, or diabetes mellitus. This review will examine the pathological and biochemical processes of atherosclerotic plaque formation and growth, with particular focus on the aging risk vis-a-vis arterial homeostasis. Particular focus will be placed on the impact of a number of important contributors to arterial homeostasis including bone marrow (BM)-derived vascular progenitor cells, differential monocyte subpopulations, and the role of cellular senescence. Finally, this review will explore many critical observations in the way the disease process has been reassessed both by clinicians and researchers, and will highlight recent advances in this field that have provided a greater understanding of this aging-driven disease.

## Atherosclerosis: ancient and inflammatory

Atherosclerosis is defined by the formation and growth of plaques within the arterial lumen with concurrent loss of vascular elasticity. Eventually, this condition can lead to consequent blood flow reduction through the affected vessel, and has long been associated with CVD-related death. Around the year 1505, Leonardo da Vinci recorded his observations of a “parched and shrunk and withered … artery that feeds the heart,” attributing this as the cause of death of an elderly man who passed away “without any movement or sign of anything amiss” (McCurdy, 1923). However, CVD remained a relatively uncommon cause of death until the dramatic surge in CVD mortality in the past 100 years, due primarily to marked and progressive increases in life expectancy, as a result of improved sanitation and public health, with a staggering reduction in communicable diseases and violent deaths. During this time period, numerous traditional lifestyle-associated CVD risk factors were identified and increased in prevalence, leading many to believe that atherosclerosis is an exclusively lifestyle-dependent modern disease. However, recent reports have challenged this view, demonstrating that mummies from various ancient societies—from as much as 5,300 years ago—exhibited a high prevalence of arterial changes compatible with atherosclerosis (Thompson et al., 2013; Clarke et al., 2014; Thomas et al., 2014; Zink et al., 2014). Additionally, a single nucleotide polymorphism (SNP) recently shown to be a genetic risk factor for CVD (minor allele of rs10757274 on chromosome 9p21) was detected in at least one of these mummies, despite the fact that it was initially believed that this gene modification arose much later in history (Zink et al., 2014). Many putative mechanisms have been considered to account for the presence of CVD that far back in human history (Thompson et al., 2013; Thomas et al., 2014). In contrast, risk factors most commonly associated with disease in modern populations include modifiable risks—such as tobacco use, obesity, diet, and physical inactivity—and non-modifiable risks—including age, gender, numerous identified SNPs in germ cells, and race (Yusuf et al., 2004; Goldschmidt-Clermont et al., 2012). Algorithms designed to interpret these factors and provide relative individual risk scores [Framingham (D'Agostino et al., 2008), QRISK®2-2016 (Hippisley-Cox et al., 2008), ACC/AHA ASCVD Goff et al., 2014] have concluded that the strongest risk factor for atherosclerosis and CVD is indisputably age; using existing algorithms, simply increasing age—while holding all other variables constant—results in a marked increase in risk for CVD (Rauscher et al., 2003; Karra et al., 2005; Goldschmidt-Clermont et al., 2012; Madonna et al., 2016). The average annual rates of first CVD events for men increase over 20-fold or more between the age groups 35–44 (incidence of 3 per 1,000) and 85–94 (incidence of 74 per 1,000), while similar rates are observed in women ~10 years later in life (Benjamin et al., 2017). Similarly, the average age of myocardial infarction is 65 and 72 for men and women, respectively (Members et al., 2016). These acute clinical events occurring at advanced ages often manifest in the absence of associated symptoms, and are frequently fatal (Myerburg and Junttila, 2012). However, atherosclerosis itself—as the principal mechanism for the majority of these events—is a chronic, inflammatory, progressive disease with initial manifestations beginning at young ages (McGill et al., 2000; Zieske et al., 2002; Hong, 2010). Evidence of the disease has been reported in multiple arterial regions in children under the age of 10, and in the aortas of individuals less than a year old (McGill et al., 2000; Hong, 2010). Advanced lesions of atherosclerosis have been detected in the arteries, including coronaries, of US military even at a young age (Enos et al., 1953; Webber et al., 2012). Importantly, although the results of these studies may not be directly comparable to the general population for reasons such as risk factor disparity between groups (notably smoking and obesity), they provide two important insights to disease prevalence over the past several decades: first, that disease prevalence has appreciably declined during this time, and, second, that onset of disease occurs at an early age and has potential to progress rapidly with age (Levy, 2012).

Historically, atherosclerosis was often viewed exclusively as the result of dyslipidemia. Both high-density lipoprotein (HDL) and low-density lipoprotein (LDL) play critical roles in the transport of cholesterol and have been implicated in atherosclerosis (Fisher et al., 2012; Ference et al., 2017). Specifically, normal levels of HDL and HDL-cholesterol (HDL-C)—often called the “good cholesterol”—are associated with a variety of antiatherogenic processes and reduced levels of CVD overall (Fisher et al., 2012; Rosenson et al., 2016). In contrast, elevated levels of LDL and LDL-cholesterol (LDL-C) have been implicated in atherosclerosis progression (Fisher et al., 2012; Ference et al., 2017). However, we now know that elevated circulating LDL alone is generally insufficient to account for the observed extent of disease and is responsible for at most half of arterial lesions of atherosclerosis in elderly people, while disease can be found even in the complete absence of hyperlipidemia (Libby et al., 2009; Wick and Grundtman, 2012). Near the end of the twentieth century, the notion that atherosclerosis was a fundamentally inflammatory disease began to gain popularity, catalyzed largely by pathologist Russel Ross' pioneering report, “Atherosclerosis—An Inflammatory Disease,” and by the work of Peter Libby (Ross, 1999; Libby, 2002; Libby et al., 2002). The formation and progression of atherosclerotic plaques as we understand today largely support Ross' own “response-to-injury” hypothesis, implicating plaque formation as a consequence of focal endothelial cell (EC) injury and inflammation (Tuttolomondo et al., 2012; Wick and Grundtman, 2012). However, as more of the underlying mechanisms and pathways controlling atherosclerosis are identified, it becomes clear that this disease does not simply follow a single, forward direction of irreversible progression. In contrast, it is evident now that atherosclerosis is the result of a myriad of independent pathways and their complex interactions (Libby, 2016). These pathways involve a multitude of various players, including various subpopulations of immune competent cells such as monocytes and macrophages, bone marrow (BM)-derived progenitor cells, chemokines and receptors, and cellular and subcellular fates including protein aggregation, cellular senescence, and apoptosis. In the following sections, each of these various aspects of atherosclerosis—and the ever-present effect of aging on these pathways and players, and beyond—will be explored.

## Arterial homeostasis, aging, and balance between injury and repair

Recently, it has been shown that genetic expression profiles and epigenetic modifications exert complex control and regulatory mechanisms for atherosclerosis (Karra et al., 2005; Dunn et al., 2014). Perhaps most critical to the understanding of this disease, are the recent findings that the most determinant factors for atherosclerosis progression are repair mechanisms, slowing or preventing the progression of disease, thus maintaining arterial homeostasis in spite of the presence of risk factors (Goldschmidt-Clermont, 2003). Specifically, a growing body of evidence indicates that vascular repair, effected by BM-derived competent vascular progenitor cells that are programmed by chemokines, cytokines, matrix proteins, and growth factors, serves to counter the effects of endothelial injury and dysfunction that would otherwise lead to atherosclerosis progression (Rauscher et al., 2003; Karra et al., 2005; Madonna et al., 2016). Using a well-established mouse model of atherosclerosis (severely hyperlipidemic, apolipoprotein E-deficient mice), it was shown that with aging, the capacity of BM-derived progenitor cells to repair arteries damaged by high lipid concentrations becomes impaired (Rauscher et al., 2003), and that the development of atherosclerotic lesions occurs contemporaneously with the loss of repair capacity, and not simply when the severe hyperlipidemia begins (Karra et al., 2005). Other work has identified the accumulation of senescent cells that produce pro-inflammatory cytokines and matrix metalloproteinases (MMPs) within atherosclerotic sites, but not in adjacent surrounding tissues (Childs et al., 2016). Identification of these cells is generally dependent on the presence of p16^INK4A^, a cyclin-dependent kinase inhibitor responsible for cell cycle arrest in senescent cells (Baker et al., 2011, 2016). The factors released by senescent cells constitute a senescence-associated secretory phenotype (SASP), and have been shown to propagate atherosclerosis, while the selective targeting and elimination of these senescent cells (referred to as senolysis) has been shown to slow atherosclerotic lesions growth through reduction of inflammatory and adhesion factors (Childs et al., 2017). Additional data suggests that BM-derived progenitor cells may protect from these effects through reduction of endothelial senescence (Rauscher et al., 2003; Baker et al., 2016; Childs et al., 2016). Another interesting potential source of arterial aging to have emerged only recently involves the formation and accumulation of improperly folded proteins (Ayyadevara et al., 2016). Although most commonly associated with neurodegenerative diseases such as Alzheimer's and Parkinson's disease, recent reports have indicated that dysfunctional processes of either protein production or degradation (resulting in aggregation) lead to proteotoxicity and have been linked to CVD (Willis and Patterson, 2013). Additional reports have confirmed the presence of upregulated protein aggregates in models of aging and hypertension, identifying proteins within these aggregates known to be involved with CVD, and suggesting a variety of causes for aggregation including cellular senescence (Ayyadevara et al., 2016). These findings may indicate a novel potential therapeutic target by examining the processes by which these protein aggregates form. Taken together, these results suggest that methods aimed at improving vascular repair, as an additional strategy to that of reducing vascular injury, may provide significantly heightened results in improving arterial homeostasis.

More recently, the crucial role of the BM in the maintenance of arterial homeostasis was further evidenced. Indeed, a novel impact of aging as a driver of cardiovascular disease risk was identified within hematopoietic progenitor cells. With aging, these cells have been shown to acquire somatic DNA mutations that provide competitive growth advantages and lead to an expanded pool of mutated clones (a process known as clonal hematopoiesis of intermediate potential, or CHIP; Steensma et al., 2015). A recent study examined over 8,000 individuals from case-control studies, and revealed that elderly carriers of CHIP had nearly double the risk of coronary heart disease when compared to non-carriers (Jaiswal et al., 2017). Moreover, this study demonstrated that the loss of function mutation of Tet2 (the second most commonly mutated gene in CHIP) led to accelerated atherosclerosis in mice, likely as a result of transcriptional modifications of associated macrophages (Jaiswal et al., 2017). Interestingly, the loss of Tet2 also resulted in a highly induced expression of interleukin-1 beta (IL-1β), a well-characterized proinflammatory cytokine known to be involved in the development of atherosclerotic plaques (Jaiswal et al., 2017; Ridker et al., 2017). IL-1β is also activated via inflammasomes containing NOD-like receptors (NLRs; Furman et al., 2017; Ridker et al., 2017). Moreover, examination of inflammasome expression profiles revealed that individuals with profiles resulting in constitutive expression of IL-1β present characteristics (arterial stiffness, elevated blood pressure, oxidative stress, and chronic levels of inflammatory cytokines) associated with CVD, while inhibition of IL-1β resulted in significant reductions in recurrent CVD (Furman et al., 2017; Ridker et al., 2017). In addition to Tet2, mutated genes identified in this work include *DNMT3A, ASXL1*, and *JAK2*, and are associated with various processes including DNA methylation, chromatin remodeling, and cell proliferation. In similar fashion, other reports have demonstrated the critical importance of epigenetic modification of various genes involved in atherosclerosis. Specifically, it was shown that the methylation of CpG islands in the promoter region of the gene encoding for estrogen receptor alpha (ERα) is not only strongly associated with aging, but is responsible for the proliferation of smooth muscle cells involved in the growth of atherosclerotic plaques (Post et al., 1999; Ying et al., 2000).

Another critical area of interest with regards to aging and arterial homeostasis as it pertains to atherosclerosis is that of specific monocyte subpopulations. As recently as the late 1980s, it was widely believed that monocytes presented as a single population of cells (Stansfield and Ingram, 2015). However, it was more recently demonstrated that these cells represent a heterogeneous mixture of cell subpopulations with distinct patterns in surface receptor expression as well as primary function in response to conditions such as atherosclerosis (Gordon and Taylor, 2005). In mice, these subsets are often characterized as being either “inflammatory” (Ly6C^hi^) monocytes that play an innate roles in antimicrobial defense, or “resident” (Ly6C^low^) monocytes that actively patrol lumenal surface of the vasculature, removing damaged cells and debris, and generally promoting arterial homeostasis (Geissmann et al., 2003; Woollard and Geissmann, 2010; Thomas et al., 2015). This patrolling behavior was first observed in the small vessels of the microcirculation, but has since been observed in larger vessels such as the carotid artery (Geissmann et al., 2003; Quintar et al., 2017). Importantly, it was shown that this patrolling behavior plays a potential role in endothelial protection, and that atherosclerosis can trigger an upregulation of the number of this subpopulation of monocytes (Quintar et al., 2017). In humans, monocyte subpopulations are divided into three main categories, and share many characteristics with those identified in mice: the “classical” or “inflammatory” monocytes (CD14^++^CD16^−^), the “non-classical” monocytes (CD14^+^CD16^++^) involved in arterial patrolling, and the more recently identified “intermediate” (CD14^++^CD16^+^) subpopulation of monocytes (Passlick et al., 1989; Geissmann et al., 2003; Woollard and Geissmann, 2010; Ziegler-Heitbrock et al., 2010; Stansfield and Ingram, 2015). Human studies of atherosclerosis have mirrored those in mice, demonstrating that non-classical CD16^+^ monocytes are primarily associated with disease, and further suggest roles for monocyte patrolling in injury healing and homeostasis (Stansfield and Ingram, 2015). However, additional monocyte subpopulations have also been implicated in atherosclerosis progression in both mice and humans (Hilgendorf et al., 2015; Stansfield and Ingram, 2015). For example, although some results have shown that intermediate monocyte levels serve as an independent predictor of acute cardiovascular events such as stroke and myocardial infarction, additional population studies have demonstrated that increased levels of circulating classical monocytes serve as a reliable predictor of cardiovascular events regardless of age, sex, or other known traditional risk factors (Hilgendorf et al., 2015). Additionally, classical Ly6C^hi^ monocytes in mice were shown to infiltrate the endothelium at sites of injury, and contribute to foam cell formation (Swirski et al., 2007; Stansfield and Ingram, 2015). Ultimately, each of these subpopulations play varying roles in the formation, progression, and also regression of atherosclerosis.

As our understanding of this complex disease advances, it has become clear that there are numerous competing forces of disease progression and repair. It appears likely therefore that disease progression is the result of both the accumulation of signals and processes which exacerbate atherosclerotic inflammation, and which limit the efficiency of repair mechanisms, as a consequence of aging and exposure to traditional risk factors (Rauscher et al., 2003; Goldschmidt-Clermont et al., 2012). Given these recent advances in the understanding of this disease, it is important to review the proposed process by which arterial plaques form, and the importance of the competing forces of inflammation and repair throughout.

## Formation of the atherosclerotic plaque: local and distant (especially bone marrow function) modifiers

**Figure 1:**
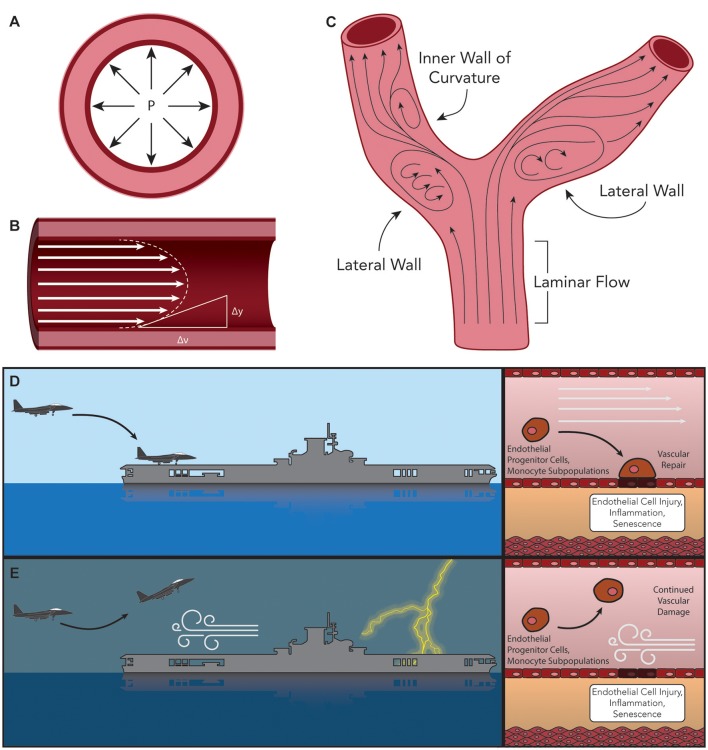
Hemodynamic forces exerted on the arteries. **(A)** Trans-mural pressure exerted radially across the vessel wall. **(B)** Endothelial Shear Stress (ESS), determined as the product of blood viscosity (η) and its shear rate (^δ*v*^/_δ*y*_) such that ESS = η • (^δ*v*^/_δ*y*_). **(C)** Typical sites of atherosclerotic plaque formation (lateral walls of bifurcations, inner wall of vessel curvature, and near branch points). Laminar flow in these regions is disrupted, causing oscillatory or reversed flow and subsequent low ESS. Comparison of the ability of bone marrow (BM)-derived vascular progenitor cells and other reparative factors to interact with injured endothelial cells under **(D)** laminar or **(E)** turbulent flow can be envisioned as the difference between landing a plane under calm or stormy conditions, respectively.

Although risk factors for atherosclerotic disease systemically affect all regions of the vasculature, plaque formation is not uniformly distributed. Instead, atherosclerotic plaques typically occur at a number of reproducible focal sites such as the inner wall of vessel curvature, the lateral walls of bifurcations, and near branch points (Pan, 2009; Warboys et al., 2011). An elegant theory to explain this plaque distribution disparity relies on the relative amount of shear stress exerted on the ECs in contact with the blood as it traverses the dynamic lumen geometry. There are two dominant forces exerted by blood on the inner lining of ECs within the vasculature. These hemodynamic forces include transmural pressure (a force exerted radially across the vessel wall, Figure 1A) and shear stress (the frictional force applied to the ECs by blood tangential to the direction of flow, Figure 1B). The value of endothelial shear stress (ESS, sometimes called wall shear stress or WSS) is determined as a function of blood viscosity (η) and its shear rate (^δ*v*^/_δ*y*_, a measure of the rate at which blood velocity increases from the endothelium toward the center of the vessel, Figure 1B). Blood flow in regions of relatively straight, uniform diameter vessels exhibits nearly laminar flow, resulting in ESS-values of 20–70 dyne/cm^2^ (2–7 Pa) (Chatzizisis et al., 2007; Chiu and Chien, 2011). However, at the aforementioned locations prone to atherosclerotic plaque formation, the laminar flow characteristics are disrupted, causing regions of oscillatory or reversed flow and subsequent reduction in ESS (<10 dyne/cm^2^, Figure 1C). In these regions of disturbed flow, a number of events and responses to low shear stress occur that contribute to the process of atherosclerotic plaque formation.

**Figure 2:**
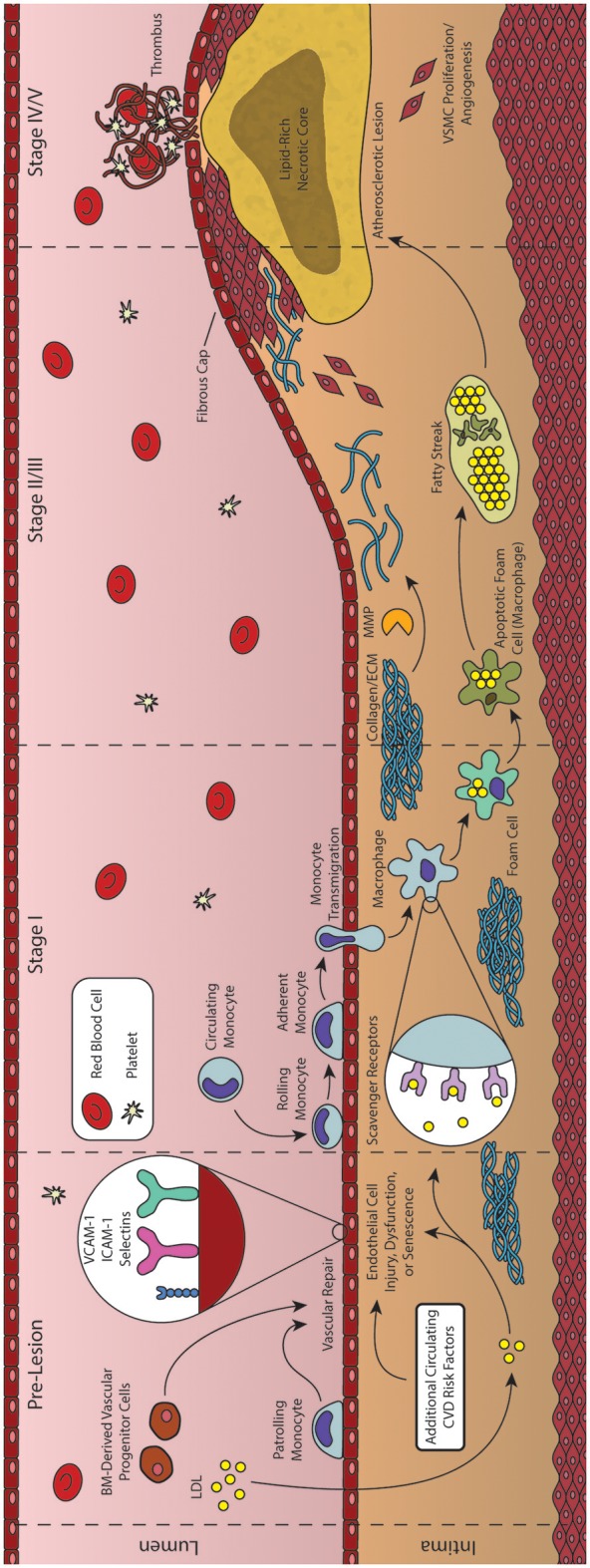
Formation and progression of an atherosclerotic plaque. As an inflammatory disease, the initial stages of atherosclerosis involve an inflammatory insult to the endothelial cells lining the artery lumen. Bone marrow (BM)-derived progenitor cells have been shown to be critical in responding to vascular injury, effecting vascular repair and maintaining homeostasis. In the absence of vascular repair, injured endothelial cells begin to express adhesion molecules that facilitate the transmigration of monocytes into the vessel intima. These monocytes then differentiate into macrophages and begin engulfing lipid and lipid products, forming foam cells. As foam cells aggregate, they form the characteristic fatty streak, while many macrophages begin to undergo apoptosis. Inefficient clearance of apoptotic macrophages leads to secondary necrosis, resulting in a growing lipid-rich necrotic core. In response to the growing lesion, smooth muscle cells migrate to the intima, helping to form the overlying fibrous cap. Rupture of this cap can expose the necrotic core, leading to thrombus formation and subsequent acute cardiovascular events.

Following the “response-to-injury” hypothesis of atherosclerosis formation and progression, conditions of low overall ESS have been shown to facilitate additional insults to the endothelium leading to dysfunction. One such path initiated by low ESS is the sustained activation of sterol regulatory elements binding proteins (SREBPs). Once activated, these endoplasmic reticulum (ER)-bound transcription factors contribute to a strong inflammatory response and increase the expression of genes that encode for the LDL receptor (LDLR) (Liu et al., 2002; Xiao et al., 2013). In addition, a large volume of research has described the involvement of the lectin-like oxidized-LDL (ox-LDL) receptor-1 (LOX-1) in the initiation and progression of atherosclerosis (Mehta et al., 2006; Mitra et al., 2011; Pirillo et al., 2013). LOX-1 has been reported to be responsible for the uptake and degradation of ox-LDL, and its expression has been shown *in vitro* to be upregulated by a number of atherosclerosis-related stimuli including shear stress, inflammatory cytokines [such as IL-1β and tumor necrosis factor alpha (TNFα)], and conditions such as hypertension (Mitra et al., 2011; Pirillo et al., 2013). Moreover, it was demonstrated that the deletion of LOX-1 reduced atherosclerosis in a LDLR knockout mouse model of disease (Mehta et al., 2007). Yet another change associated with low ESS is the shift in EC morphology from tightly packed cells aligned with the direction of blood flow to a damaged morphology with cuboidal cells demonstrating no observed preferential alignment, and resulting in leakier junctions between cells (Davies, 2009; Pan, 2009; Linton et al., 2015). This disorganized cell morphology is compounded by the loss of repair mechanisms affected by progenitor cells. As discussed previously, BM-derived progenitor repair cells play a critical role in protecting arteries against atherosclerosis. One reparative role played by these cells is the restoration of damaged endothelial tissue or engraftment into these damaged regions to reverse endothelial injury (Madonna et al., 2016). Turbulent flow may disrupt the interaction between repair-competent cells in the blood and the arterial wall, thus limiting the effectiveness of the repair process (Figures 1D,E) (Xu, 2009; Chiu and Chien, 2011). It is also possible that low ESS prevents the wash-out of senescent ECs and macrophages, thus hindering their replacement by fresh such cells (Childs et al., 2016). The net result of the up-regulation of LDLR and the increased cell permeability is a dramatic increase in levels of LDL infiltration in regions of low ESS (Figure 2, Stage I) (Chatzizisis et al., 2007). Additional pro-inflammatory pathways activated under conditions of low ESS include the mitogen-activated protein (MAP) kinase and nuclear factor κB (NF-κB) signaling pathways, while levels of miR-10a and its regulatory, anti-inflammatory effects on the NF-κB pathway are attenuated under these conditions (Fang et al., 2010; Bryan et al., 2014). Recently, this strictly dichotomous view of “low” and “high” ESS and its effect on the endothelium and atherosclerosis has been revisited, and it is now suggested that—while physiological values of ESS appear to be atheroprotective—both “low” and “high” shear stress outside this physiological range may lead to atherosclerosis progression (Hung et al., 2015; Eshtehardi and Teng, 2016).

The combined net result of declining vascular repair mechanisms coupled with endothelial inflammation and the activation of ECs is the increased capture of circulating monocytes. This process, known as the leukocyte adhesion cascade, begins with the interaction of monocytes and displayed adhesion molecules in the regions of inflammation, leading to the stepwise rolling, firm adhesion, and transmigration of the monocyte into the vascular intima (Figure 2, Stage II) (Ley et al., 2007; Gerhardt and Ley, 2015). Monocyte recruitment is largely directed by the presence of EC-derived chemokines (including CCL5, CCL2, and CX_3_CL1), their respective receptors (including CCR2, CXCR3, CX_3_CR1, and others), and various adhesion molecules such as VCAM-1 and ICAM-1 (Geissmann et al., 2003; Tacke et al., 2007; Tuttolomondo et al., 2012; van der Vorst et al., 2015). Following transmigration from the lumen into the intima, these monocytes undergo differentiation into macrophages capable of engulfing the growing pool of apoB-containing LDL and its modified variants (Ley et al., 2011). These differentiated macrophages identify and engulf LDL through expression of scavenger receptors such as oxidized LDL receptor 1 (LOX-1), scavenger receptor A (SRA), and CD36 (Figure 2, Stage II) (Yoshida and Kisugi, 2010; Ley et al., 2011).

Instructively, through the use of CD36/SRA double knockout mice, it has been shown that lipid-laden macrophages will still accumulate in atherosclerotic vessel walls, suggesting additional routes of lipid uptake (Manning-Tobin et al., 2009). Other proposed mechanisms include the capability of macrophages to accumulate LDL via receptor-independent pinocytosis, and the hydrolysis of free cholesterol from LDL aggregates (Barthwal et al., 2013; Bentzon et al., 2014). Cholesterol liberated from lipoproteins taken up by these pathways is transported within the macrophage to the ER where it is esterified to cholesteryl esters by acetyl-coenzyme A:cholesterol acetyltransferase 1 (ACAT1) and neutral cholesterol ester hydrolase (nCEH), while reverse cholesterol transport is controlled by ATP-binding cassette (ABC) transporters including ABCA1 and ABCG1 (Ley et al., 2011). Under healthy conditions, the liberated cholesterol is accepted by HDL particles primary via protein ApoA1, while modification of this protein can produce dysfunctional HDL and produce proatherogenic effects (Fisher et al., 2012). However, under atherosclerotic conditions, macrophages demonstrate both an increase in lipid uptake and inefficient efflux of cholesteryl esters, generating characteristic lipid-laden foam cells (Figure 2, Stage II) (Yu et al., 2013). Agonists for the Liver X Receptor (LXR) transcription factors have been proposed as potential therapeutic agents to improve cholesterol efflux and thus reduce foam cell formation, but have also been shown to induce liver steatosis (Levin et al., 2005; Hijmans et al., 2015). Moreover, while efforts have been made to generate synthetic agonists avoiding this effect, recent results have suggested that these agents do not function directly through cholesterol efflux (Kratzer et al., 2009; Kappus et al., 2014). It has been shown that such defects in lipid metabolism can cause ER stress and dysfunction, ultimately leading to apoptosis of the cell (Seimon et al., 2010). Compounding this problem, defects in cholesterol efflux also inhibit efficient efferocytosis (clearance of apoptotic cells by nearby macrophages), leading to an accumulation of apoptotic cells in these regions. The growing population of apoptotic macrophages and foam cells generate the characteristic fatty streak associated with early atherosclerotic plaques (Figure 2, Stage III/IV) (Moore and Tabas, 2011; Van Vré et al., 2012). Apoptotic cells not cleared through phagocytosis then undergo secondary necrosis, releasing cellular debris along with additional oxidized lipids and pro-inflammatory cytokines that contribute to continued inflammation and a growing necrotic core of the more advanced atherosclerotic lesion (Figure 2, Stage IV) (Moore and Tabas, 2011; Bentzon et al., 2014).

Atherogenesis often ceases here, with many lesions progressing no further than the fatty streak, due in large part to various reparative mechanisms such as those initiated by BM-derived progenitor cells (Goldschmidt-Clermont et al., 2012). However, some lesions undergo additional changes within the vascular intima causing a pronounced growth of the plaque and remodeling of the vessel itself. Specifically, macrophages and foam cells within the fatty streak begin releasing inflammatory cytokines (IL-1β, IL-4, and TNFα) that interact with various growth factors [platelet-derived growth factor (PDGF), CD-40] to activate MMPs (Chistiakov et al., 2013). Interestingly, the role of IL-4 has been debated, with early studies demonstrating that IL-4 deficiency in a mouse model of atherosclerosis reduced lesion formation, while later studies found a lack of involvement of this cytokine regardless of disease induction method (Ramji and Davies, 2015). Once activated, these MMPs are responsible for remodeling and weakening the extracellular matrix (ECM) surrounding the vascular smooth muscle cells (VSMC) of the vessel. As a result of the weakened ECM, arterial cells begin to sense transmural pressure as a micro-angioplasty stretch with each pulse, stimulating growth factor release by components of the fatty streak that promote migration of these stretched VSMCs to the growing lesion (Rudijanto, 2007). Under healthy physiological conditions, mature VSMCs rarely undergo proliferation or migration, and exhibit a contractile phenotype (expressing α-smooth muscle actin and calponin) in which they control blood vessel diameter and blood flow through vasodilation or vasoconstriction (Rudijanto, 2007; Zhang et al., 2016). However, in response to injury, VSMCs undergo a transition to a non-contractile or synthetic phenotype, decreasing contractile marker expression, and generating collagen-rich fibrous ECM that eventually represents a major component of this newly formed fibroatheroma (Figure 2, Stage V) (Bentzon et al., 2014; Zhang et al., 2016). Complicating this process further, neither elevated levels of cholesterol nor extensive macrophage involvement are strictly required for this VSMC proliferation. As discussed previously, other mechanisms—such as epigenetic modification of specific genes associated with SMC proliferation—have been shown to be involved in the progression of atherosclerotic plaques (Post et al., 1999; Ying et al., 2000). In addition, deposition and growth of calcium granules may also occur, and has been shown to exhibit both stabilizing and destabilizing effects to the vulnerability of the fibrous cap depending on their extent and location (Li et al., 2007). Indeed vascular calcification, a condition pathognomonic for atherosclerosis, may be quantified in the coronary arteries using computed tomography (CT) to generate a coronary artery calcium (CAC) score that is used in conjunction with the Framingham Risk Score to improve prognostic value of coronary events (Yeboah et al., 2012; Maurovich-Horvat et al., 2014). Modulations of the ECM and proliferation of VSMCs are also required for arterial remodeling, which can be either constrictive or expansive (as a compensatory mechanism to counter atherosclerotic plaque growth) (Wentzel et al., 2012; Bentzon et al., 2014). These advanced plaques also possess the potential to undergo rupture or erosion, exposing the thrombogenic core to the blood flow of the vessel lumen (Figure 2, Stage VI). A common trait of plaques vulnerable to rupture is a thin covering of ECs, VSMCs, and ECM—termed thin-cap fibroatheromas (TFCAs) and possessing a cap of <65 μm in thickness (Virmani et al., 2000). Activation of MMPs from abundant macrophages present in the plaque may cause degradation of the ECM near the cap, destabilizing EC adhesion and allowing for exposure of the necrotic core, leading to subsequent thrombus formation in the lumen of the vessel. Although this is a rare fate for atherosclerotic plaques (most either do not progress to advanced stages or become stable plaques with caps >65 μm), the subsequent formation of a thrombus from plaque rupture can cause complete occlusion of the local artery, with consequent myocardial infarction and/or sudden cardiac death. Another potential thrombogenic fate of an atherosclerotic plaque is that of plaque erosion, in which a thrombus is formed in the absence of rupture, often with a disrupted endothelium and an enhanced proliferation of VSMCs (Virmani et al., 1999; Braunwald, 2013). Although less common than plaque rupture, plaque erosion has been shown to be more common in women and the elderly, while the underlying mechanisms of plaque erosion are only beginning to be elucidated and still incompletely understood (Libby and Pasterkamp, 2015; Chandran et al., 2017). Regardless of the etiology of the plaque source, the resulting thrombus formed by these processes may also embolize and travel to a secondary vessel causing ischemia, myocardial infarction, or sudden coronary death (Davies, 2000; Bentzon et al., 2014). Alternatively, repeated cycles of clinically silent rupture of the cap followed by healing may contribute to overall plaque growth and luminal stenosis. Another potent source of plaque growth is intraplaque hemorrhage. As the atherosclerotic plaque grows, the region becomes progressively more inflamed as well as hypoxic, inducing angiogenesis and vasa vasorum into the plaque itself. These neovessels have been observed to be fragile and leaky, and are an important source of hemorrhage within the wall of the artery, contributing not only to further inflammation, but also plaque growth and vessel stenosis (Kolodgie et al., 2003). In this way, tissue ischemia caused by luminal stenosis can occur even in the absence of clinically significant rupture (Kovacic and Fuster, 2012).

## Control of inflammation: a novel therapeutic opportunity to restore arterial homeostasis

**Figure 3:**
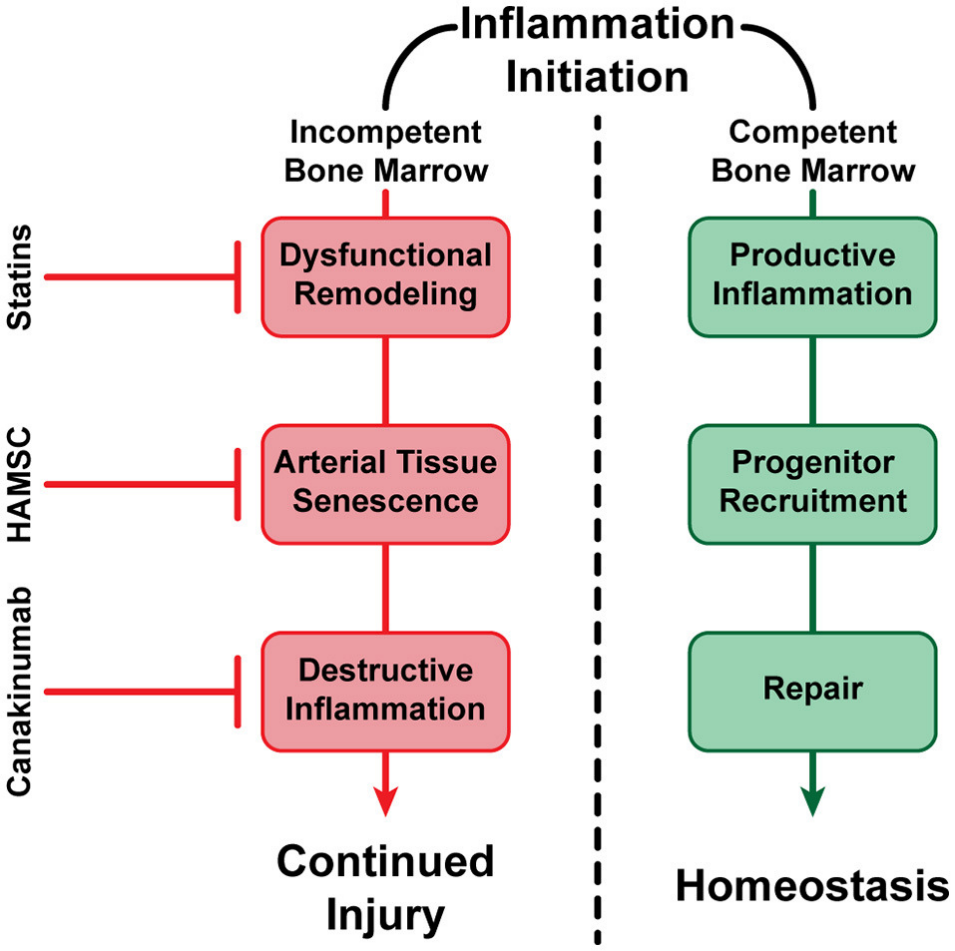
A visualization of alternative fates of vascular inflammation. In the presence of healthy BM-derived vascular progenitor cells capable of arterial repair, inflammation subsides and vanishes (negative feedback loop), resulting in the maintenance of vascular homeostasis. In the absence of sufficient BM-derived vascular progenitor cells, or if such cells are no longer capable of arterial repair, inflammation continues or even expands, resulting in potential dysfunctional remodeling, continued cell senescence, and propagation of arterial injury (positive feedback loop). However, successful treatments have been established such as statin therapy, which has been shown to reduce circulating LDL-Cholesterol (LDL-C), increase HDL-Cholesterol (HDL-C), reducing inflammation and dysfunctional remodeling (Nozue et al., 2013); Human Allogeneic Mesenchymal Stem Cells (HAMSC) (Tompkins et al., 2017), and possibly other progenitor cells (Song et al., 2012), may be able to reverse arterial tissue senescence and improve arterial repair without affecting circulating lipids; and anti-inflammatory molecules such as Canakinumab have also been shown to reduce destructive inflammation without affecting circulating lipids (Ridker et al., 2017). Hence, our armamentarium to prevent, stabilize or even reverse atherosclerosis and its thromboembolic complications are becoming increasingly effective at promoting arterial homeostasis.

Atherosclerosis is the most significant human health problem globally. We know today that the disease does not follow a simple, unidirectional progression, and is determined by a myriad of pathways, control mechanisms, and repair processes; these encompass multiple inflammatory molecules, BM-derived progenitor cells, a range of immune cells such as specific monocyte subpopulations, genetic mutations, and epigenetic modifications among numerous other participants both known and those yet to be discovered. Ultimately, however, the clinical result for an immensely large number of individuals is the formation and growth of vascular lesions with the potential to rupture, leading to life-threatening conditions. It is imperative to continue to evolve technological strategies to both predict and detect the formation, progression, and clinical status of these atherosclerotic plaques, while additional details are elucidated regarding the process of disease progression. One example of important progress has been made in the control of inflammation when inflammation is no longer promoting repair, but instead has taken a damaging role for the artery. It was recently reported that a monoclonal antibody against IL1-beta, when injected systemically to patient with CVD and high inflammatory index (CRP levels ≥ 2 mg/mL), is capable of reducing risk for coronary events, even with already reduced lipids and had no further effect on lipid levels (Ridker et al., 2017). The role of inflammation is increasingly established in the progression of arterial lesions, and it is useful to consider inflammation in the context of arterial homeostasis. Arterial repair is triggered and controlled by molecules that belong to inflammatory pathways. However, as was hypothesized and subsequently demonstrated in an animal model, the progression of atherosclerotic inflammation is modulated by the presence or the absence of an efficient repair process (Goldschmidt-Clermont et al., 2005). In the presence of BM vascular progenitor cells capable of arterial repair, the artery heals and inflammatory signals subside and vanish. However, as previously discussed, reductions in the availability of BM-derived vascular progenitor cells occurring as a consequence of aging or genetic susceptibility (exhaustion or dysfunction) result in a lack of arterial healing. These reductions can occur either because repair-capable cells are no longer produced effectively by the BM, because the produced cells have become dysfunctional, or a combination thereof. Consequently, inflammatory signals do not subside and vanish, and indeed are heightened to the point where they attract and support monocytes/macrophages and other immune competent cells that further enhance arterial injury (Figure 3). Hence, the maintenance of arterial homeostasis is a complex process that must balance injuries to the arterial wall (lipids, smoke, etc.), inflammatory processes required for triggering and supporting arterial repair, and the renewal of BM-derived vascular progenitor cells that are necessary for such repair.

The example of inflammation suppression in individuals with established CVD is important, as it proves that inflammation can contribute to the disease, and that the control of excessive inflammation via specific agents can lead to consequent reduction in CVD complications. However, this work addresses only one aspect of the overwhelmingly complex disease that is atherosclerosis. As discussed herein, the pathways and players involved in the regulation and progression of this ubiquitous disease are immensely diverse, and subject to continuous reassessment. In much the same way that inflammation has been shown to be involved in the repair of arterial damage or contribute to disease progression when excessive, each component of atherosclerosis may play a critical part depending on the context, while many such components may not yet be fully established. It is instructive that administration of adult mesenchymal stem cells to individuals suffering from aging frailty has been shown, in double blind placebo-controlled prospective randomized trial, to improve functional outcomes of patients, many of which being dependent on the integrity of the cardiovascular system (Tompkins et al., 2017). The concurrent approaches of taming excessive inflammation with specific antagonists, and augmenting the cellular capacity for arterial repair with selected stem cells, in addition to the previously developed armamentarium, may represent the strategies required to restore arterial homeostasis whenever lost, and help to reduce atherosclerosis and its dreadful complications during the twenty-first century.

## Author contributions

All authors contributed equally to the drafting, editing, and final composition of the manuscript. TH: designed and prepared the corresponding figures for the manuscript.

### Conflict of interest statement

The authors declare that the research was conducted in the absence of any commercial or financial relationships that could be construed as a potential conflict of interest.
